# Impact of Cyanotoxin Ingestion on Liver Cancer Development Using an At-Risk Two-Staged Model of Mouse Hepatocarcinogenesis

**DOI:** 10.3390/toxins14070484

**Published:** 2022-07-14

**Authors:** Igor Mrdjen, Jiyoung Lee, Christopher M. Weghorst, Thomas J. Knobloch

**Affiliations:** 1 College of Public Health, Environmental Health Sciences, The Ohio State University, Columbus, OH 43210, USA; mrdjen.1@osu.edu (I.M.); lee.3598@osu.edu (J.L.); weghorst.2@osu.edu (C.M.W.); 2 Department of Food Science and Technology, The Ohio State University, Columbus, OH 43210, USA

**Keywords:** hepatocellular carcinoma, *Microcystis*, cancer promotion, cyanotoxin, microcystin, toxicity

## Abstract

Exposure to cyanobacterial hepatotoxins has been linked to the promotion and increased incidence of liver cancer in pre-clinical and epidemiologic studies. The family of hepatotoxins, microcystins (MCs), are produced by over 40 cyanobacterial species found in harmful algal blooms (HABs) worldwide, with MC-LR being the most common and potent MC congener. In the current study, we hypothesized that the low-dose chronic ingestion of *Microcystis* cyanotoxins via drinking water would promote liver carcinogenesis in pre-initiated mice. Four groups of C3H/HeJ mice received one intraperitoneal (i.p.) injection of diethylnitrosamine (DEN) at 4 weeks of age. Three weeks later, the mice were administered ad libitum drinking water containing one of the following: (1) reverse osmosis, deionized water; (2) water containing 500 mg/L phenobarbital (PB500); (3) water with purified MC-LR (10 µg/L) added; or (4) water containing lysed *Microcystis aeruginosa* (lysate; 10 µg/L total MCs). The exposure concentrations were based on environmentally relevant concentrations and previously established Ohio EPA recreational water MC guidelines. Throughout the 30-week exposure, mouse weights, food consumption, and water consumption were not significantly impacted by toxin ingestion. We found no significant differences in the number of gross and histopathologic liver lesion counts across the treatment groups, but we did note that the PB500 group developed lesion densities too numerous to count. Additionally, the proportion of lesions classified as hepatocellular carcinomas in the MC-LR group (44.5%; *p* < 0.05) and lysate group (55%; *p* < 0.01) was significantly higher compared to the control group (14.9%). Over the course of the study, the mice ingesting the lysate also had a significantly lower survival probability (64.4%; *p* < 0.001) compared to water (96.8%), PB500 (95.0%), and MC-LR (95.7%) exposures. Using cyanotoxin levels at common recreational water concentration levels, we demonstrate the cancer-promoting effects of a single cyanotoxin MC congener (MC-LR). Furthermore, we show enhanced hepatocarcinogenesis and significant mortality associated with combinatorial exposure to the multiple MCs and bioactive compounds present in lysed cyanobacterial cells—a scenario representative of the ingestion exposure route, such as HAB-contaminated water and food.

## 1. Introduction

The health effects associated with cyanotoxin exposure, which may occur during cyanobacterial harmful algal blooms (HABs), have been the subject of numerous studies [1,2,3]. The most common toxins formed during freshwater HABs are cyanobacterial microcystins (MCs). Microcystins are a family of hepatotoxins with over 100 congeners, the most potent and common of which is designated microcystin-LR (MC-LR) due to the presence of leucine and arginine at two variable positions within the monocyclic toxin’s structure [4]. The toxicity of MC-LR begins with its transport across cellular membranes via the organic anion-transporting polypeptides (OATPs), common transporters present in most epithelial cells. These OATPs are present in high levels in the liver, contributing to the enhanced toxicity in this organ system [5]. Once internalized into the hepatocyte, the ability of MC-LR to inhibit protein phosphatase 1 and 2A (PP1/2A) disrupts cellular structures, produces reactive oxygen species (ROS), promotes cellular survival, leads to apoptosis, and causes tissue inflammation [4,6].

Previous cell line studies have also linked exposure to cyanobacterial lysates to the inhibition of gap junction intracellular communication (GJIC), consequently impairing the bidirectional cytosolic exchange of metabolites and signaling molecules maintaining homeostasis and cellular growth [7,8,9]. GJIC disruption is associated with diseases such as diabetes, neuropathy, and cancer. Our previous acute toxicity study in mice demonstrated the ability of MC-LR exposure to cause hepatocellular apoptosis and damage, leading to mortality and liver dysfunction [10]. Overall, the generation of an oxidative stress microenvironment, the promotion of cellular proliferation, the induction of inflammation, and potential GJIC inhibition establish a cascading mechanism for hepatocarcinogenic promotion [11]. MC-LR-dependent oncotic necrosis can further compromise hepatic health and decrease liver function in impacted organisms [12].

Epidemiological studies have also investigated the contribution of MC-LR to liver disease and hepatocellular cancer incidence. A study in the Three Gorges Dam area of China found higher rates of liver cancer and childhood liver damage in areas where MCs were detected in food and water supplies [13,14]. Similarly, the rates of primary liver cancer were higher in the agricultural regions of Serbia impacted by HABs compared to those without reported HABs [15]. Further epidemiologic examinations of Serbian HABs showed that the rates of gonadal, gastric, colorectal, skin, and primary liver cancer were all associated with the presence of HABs in the surveyed regions [16]. The MC concentrations in some Serbian lakes exceeded 100 µ/L total MC, well above the World Health Organization (WHO) guideline levels [17,18]. Epidemiological studies have also found that HAB appearance was significantly related to non-alcoholic liver disease rates in the United States [19]. Echoing these findings, the International Agency for Research on Cancer (IARC) classifies MC-LR as a possible human carcinogen, while data on the carcinogenicity of other MCs remain limited [19,20]. The data from these studies demonstrate the need for accurate evaluation of the cancer-promoting ability of these naturally occurring hepatotoxins [4,21], especially in the presence of underlying predisposing health conditions.

Despite substantial investigative work, previous studies have been incomplete in addressing the knowledge gap for liver carcinogenic promotion following the low-dose chronic ingestion of microcystin cyanotoxins. Prior pre-clinical studies have used both mouse and rat experimental models focused on the controlled exposure delivery methods of intraperitoneal (i.p.) injection or oral gavage [22,23]. There is a notable lack of consensus in the reported outcomes across animal studies exploring MC carcinogenicity, largely due to the substantive variation in study design (i.e., rodent model, delivery method, dose, duration). For example, Nishiwaki-Matsushima et al. (1992) [22] found tumor promotion in Fischer rats initiated with diethylnitrosamine (DEN) and exposed to MC-LR via i.p. injection. Other studies have noted increases in neoplastic lesions and the induction of liver nodules during long-term exposures [24,25]. Labine & Milik (2015) found that exposure to 1 µg/L MC-LR did not result in cancer development in mice when an initiating chemical carcinogen was not used [26]. While IARC includes MC-LR as a possible human carcinogen, its role as a potential carcinogenic initiator or relevant tumor promoter remains unclear [20]. We hypothesized that MCs would function as poor de novo cancer initiators at low doses but could act as promoters of liver carcinogenesis even at the low concentrations allowable in recreational water sources, especially combined with other weak predisposing conditions.

The goal of the current study was to explore the role of MC-LR and a whole cyanobacterial lysate as potential tumor promoters in a two-stage mouse model of liver carcinogenesis. Our aim was to use a relevant route of exposure (water ingestion) to better simulate real-world environmental/recreational water exposure. While MC-LR is the most common and potent MC congener in freshwater HABs, it is not the only bioactive promoter present in *Microcystis* sp. that is available for release during cyanobacterial lysis. Consistent with prior studies, there is a rationale for suggesting that the combinatorial bioactives within whole lysed *Microcystis* could demonstrate increased biological impacts. In fact, prior work has shown an increased toxicity and potential GJIC inhibition in hepatocytes following the exposure of cellular lysates compared to pure toxins [9,27]. Since cyanobacterial concentrations can exceed 100,000 cells/mL during HABs, the added toxicity has been attributed to the independent, additive, or synergistic contribution of multiple MC congeners, cellular components, and endotoxins [9,27]. Such exposures are expected to occur in areas with insufficient water treatment methods and economically disadvantaged areas relying on at-home water treatment. This study aims to simulate real-world, chronic exposure conditions via the ad libitum ingestion of drinking water containing purified MC-LR or a cyanobacterial biomass lysate, thereby bypassing more artificial and potentially artefactual delivery exposure techniques (e.g., intraperitoneal injection, oral/gastric gavage).

## 2. Results

### 2.1. M. aeruginosa Lysate Characterization

The cultured *M. aeruginosa* produced a mixture of toxins predominantly consisting of MC-RR (37%), an MC-RR variant demethylated at the third amino acid position ([D-Asp3]MC-RR) (24%), with smaller quantities of MC-LR (22%) and MC-YR (17%) (Figure S1). The BMAA concentrations were below the limit of detection of the Abraxis BioScience ELISA.

### 2.2. Microcystin Adsorption by Plastic

No significant adsorption of the MC compounds to the OSU-ULAR cage drinking bottles was detected over a 1-week period simulating the animal protocol water delivery timeline (Figure S2). Nominal variations in the MC levels throughout the experiment can be attributed to the limitations of the ELISA kit method and the amplification of variations due to dilution.

### 2.3. Mouse Health Metrics and Dosing

**Figure 1:**
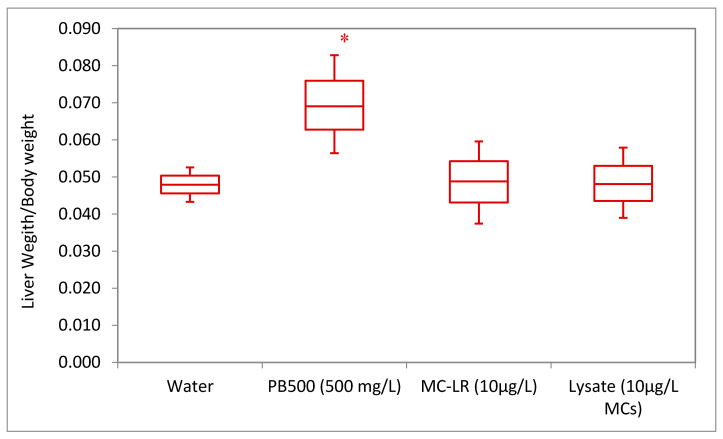
Mouse liver-weight-to-body-weight ratios by treatment group. No significant differences in the liver-weight-to-body-weight ratios were seen across the experimental treatment groups, but we did note a significantly higher (* *p* < 0.05) liver-weight-to-body-weight ratio in the PB500-positive control group.

Over the course of the experiment, the mouse body weights (Figure S3) and food consumption did not vary significantly across the treatment groups. The equivalent consumption of water over the course of the study was crucial for assessing the delivered dose and health assessment metrics, especially in the groups receiving the experimental MC treatments. The authors did note a significantly lower rate of water ingestion (*p* < 0.001) in the positive control (PB500) group (Figure S4). Due to no visual signs of decreased health status, no differences in weight, and no differences in food consumption, decreased water intake in the positive control group was likely not associated with the decreased health status of the experimental animals. Therefore, the decreased water intake was likely attributed to aversion due to the taste of the phenobarbital treatment. Further analysis of groups receiving the experimental MC treatments and the water control showed no significant differences in consumption across the groups (Figure S4). On average, the mice in the exposure groups consumed the same dose of MC-LR (1.20 ± 0.734 ng MC per gram mouse, daily) or MC equivalent (1.13 ± 0.586 ng MC per gram mouse, daily) over the course of the study. Additionally, the mouse liver-weight-to-body-weight ratios did not differ significantly across treatments (Figure 1).

### 2.4. Lesion Counts

**Figure 2:**
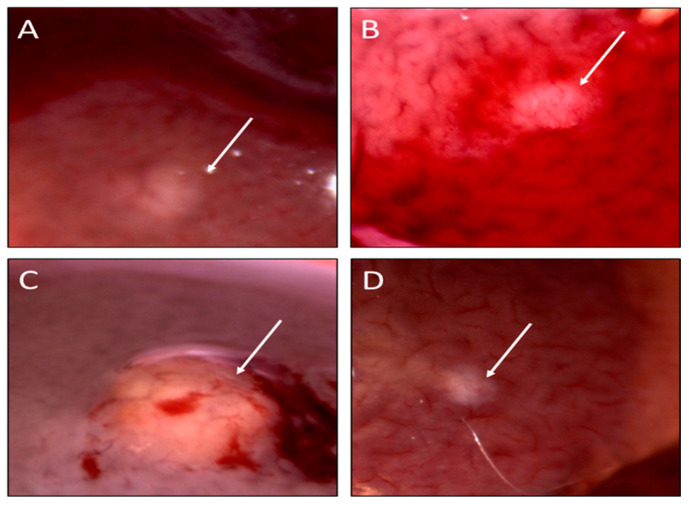
Representative gross lesions in the water-only exposure control group (**A**,**B**), lysate exposure group (**C**), and MC-LR exposure group (**D**). All groups were initiated with DEN. All lesions were imaged using an AmScope Trinocular Zoom Stereo Microscope with a 5 MP Digital Imaging System at 10× (**A**,**B**,**D**) or 20× (**C**) magnification (United Scope LLC; AmScope, Irvine, CA, USA).

**Figure 3:**
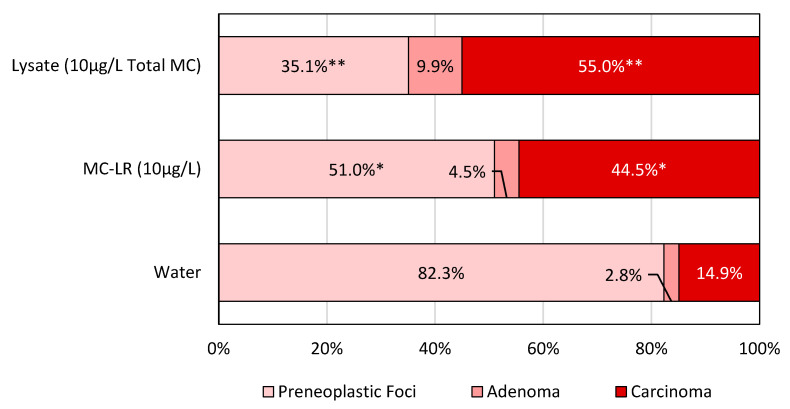
Lesion proportions by classification across treatment methods. Significance level is indicated by * (*p* < 0.05) and ** (*p* < 0.01). Histopathological lesion classification showed a significantly higher proportion of lesions classified as “carcinoma” in the lysate (55%) and MC-LR (44.5%) groups compared to the ingestion of water (14.9%). Significant differences were also noted in preneoplastic foci but not in adenoma proportions.

Lesion density within the 500 mg/L phenobarbital (PB500) liver tumor promoter positive control group was too high for gross enumeration and histopathologic classification; consequently, these data have been excluded from the comparative analyses. Excluding the positive tumor promoter control group, there were no significant differences in gross (Table 1) nor histopathologic (Table 2) lesion counts across the treatment groups. The gross examination of livers showed varying sizes and degrees of lesion development across the exposure groups (Figure 2). The histopathological lesion counts also showed no significant differences in lesion density across groups. However, the histopathological lesion classification showed a significantly higher proportion of lesions classified as “carcinoma” in the lysate (55%; *p* < 0.01) and MC-LR (44.5%; *p* < 0.05) groups, compared to the ingestion of water (14.9%) (Figure 3). Additionally, we noted that the water group presented a significantly higher proportion of preneoplastic foci than lysates (35.1%; *p* < 0.01) MC-LR (51%; *p* < 0.05). No significant differences were seen in adenoma across the groups (Figure 3).

### 2.5. Mortality

**Figure 4:**
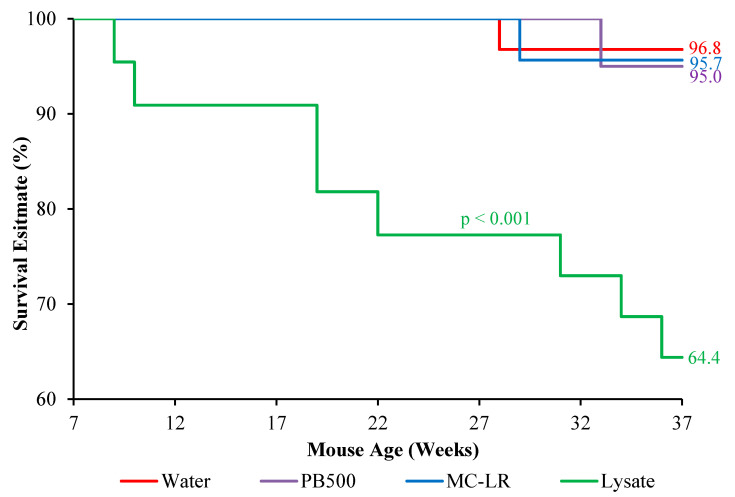
Kaplan–Meier mouse survivability estimates by exposure group. Numbers in the margins represent the survival probability at the study termination as calculated by the Kaplan–Meier curve. Chronic ingestion of the cyanobacterial lysate produced a significantly lower survival probability (64.4%) than the ingestion of PB500 (95%), MC-LR (95.7%), or water alone (96.8%).

During the study, mortality events were observed in all three groups prior to the intended protocol termination timeline (Figure 4). The experimental group ingesting the lysate demonstrated a significantly higher mortality rate (35.4%; *p* = 0.0009) than those ingesting MC-LR (4.3%) or water only (3.2%). This trend in mortality persisted steadily over the course of the experiment.

## 3. Discussion

The goal of this study was to characterize the chronic, environmentally relevant, and low-dose ingestion of *Microcystis* cyanotoxins via a drinking water route of exposure. By using MC exposure via drinking water ingestion instead of i.p. injection or oral/gastric gavage, the study more accurately simulated hazard exposure scenarios analogous to real-world environmental conditions. We noted no significant variation in food consumption, mouse weight, or water consumption (Figure S3) across the treatment groups over the course of the study, suggesting no obvious MC-associated health impacts on these measurements during the progression of DEN-induced hepatocarcinogenesis. The equivalent water consumption (matched dosing) across experimental groups, highlighted by a lack of observed hesitancy to the consumption of MC-LR or lysate-dosed drinking water, supports the continued use of ad libitum water ingestion as an appropriate delivery mode for chronic MC exposure studies.

The gross and histopathological examination of livers from the tumor promotion positive control PB500 group showed that the lesions were too numerous to quantify and classify accurately. Consequently, we have excluded the PB500 group from further discussions related to lesions and cancer progression. Excluding the PB500 group, the gross examination of mouse livers showed no significant differences in the total lesion counts across treatment methods (Table 1). Similarly, no significant changes in histopathologic lesion density normalized to liver tissue area were detected across the treatments (Table 2). However, the proportional distribution of histopathological lesions classified as carcinomas was significantly greater in the MC-LR (44.5%; *p* < 0.05) and lysate (55.0%; *p* < 0.01) treatment groups compared to the water-only exposure group (14.9%) (Figure 3). Additionally, we found that the proportion of preneoplastic foci in the MC-LR (51.0%; *p* < 0.05) and lysate (35.1%; *p* < 0.01) groups was significantly lower than that of the water group (82.3%). These findings suggest that the exposure to MC-LR or lysed *Microcystis* cellular components advances the progression of preneoplastic foci into an increasingly malignant phenotype (carcinoma). We demonstrate that chronic low doses of MCs readily attainable in recreational water sources and delivered through water ingestion can promote and shift hepatocellular lesions toward more progressed phenotypes during DEN-induced liver carcinogenesis.

**Figure 5:**
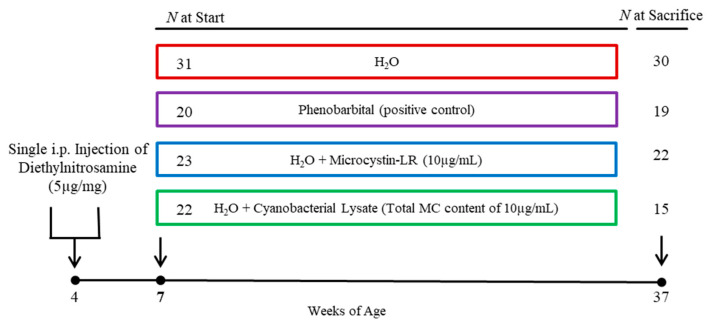
Experimental design and timeline.

Furthermore, the appearance of carcinomas in lysate-exposed mice may be underreported, as, throughout the study, we saw a significantly greater mortality rate (31.8%; *p* < 0.001) in this group compared to the MC-LR (4.3%), PB500 (5.0%), or water-only (3.3%) groups (Figure 5). These early mortalities can occur before potential lesion promotion is observable under gross examination or easily resolved with histopathological analysis. Similarly, the survival probability, as calculated via Kaplan–Meier analysis of the lysate group, was significantly lower (64.4%; *p* < 0.001) than that of the MC-LR (95.7%), PB500 (95.7%), and water groups (96.8%). The increased mortalities seen during lysate exposure were unexpected with respect to the observed equivalent food and water consumption compared to the MC-LR, PB500, and water-only groups. Consequently, the increased disproportionate mortality negatively impacted the statistical power to detect significant differences in lesion counts and distribution, impeding some intended comparisons as designed.

While others have noted an enhanced toxicity of cyanobacterial lysates compared to pure cyanotoxins, this is the first study to demonstrate such a high mortality rate using a low-dose, environmentally relevant, chronic drinking water exposure model. Chemical analysis of the *Microcystis* lysate shows that multiple MC compounds exist in the mixture, including MC-RR, MC-YR, and MC-LR. Some previous works suggest that the toxicity of such a cocktail is expected to be lower than similar concentrations of only MC-LR [28]. The presence of other common *M. aeruginosa* cyanotoxins could be excluded since the isolated strain used for this study does not synthesize anatoxin-a or saxitoxin [29].

Further potential factors causing an increase in mortality were also considered. Due to a mutation to the toll-like receptor responsible for LPS toxicity, C3H/HeJ mice are hyporesponsive to the LPS endotoxin. Not only is this mutation beneficial in lessening mortality in chronic exposure studies, but it also eliminates the attribution of LPS to mortality rates [30,31]. Therefore, the increase in toxicity seen during this study can most likely be attributed to the additive or potential synergistic toxicities of multiple MCs, pigments, acids, salts, and ROS [32]. Other studies have also postulated that increases in the toxic response may be attributed to unidentified metabolites in cyanobacteria, which have previously been shown to affect tumor promotion [9]. Additional changes in mortality could be due to the differences in toxicity and biochemical interactions across MC congeners and their derived modifications (e.g., additive toxicity across MC-LR, MC-RR, and ([D-Asp3]MC-RR). These potential differences should be investigated further in future studies, along with the biochemical composition of cyanobacterial lysates, to better understand the complex interactions of multiple available bioactive compounds.

While hepatocellular carcinomas are disproportionately observed in males compared to females—twice as common in the US and up to tenfold more common in some countries globally—further studies should incorporate both sexes [33]. While less than in the male sex, liver cancer is still a relevant risk to women. Previously, we have shown that acute, high-dose MC-LR delivered by daily gavage produced more liver toxicity in female compared to male mice [10]. Additionally, the unexpected marked increase in mortality observed in the *M. aeruginosa* lysate group necessitates increasing group sizes to safeguard the intended statistical power and to ensure that subsequent analysis appropriately interrogates the primary outcomes of lesion incidence, multiplicity, and classification.

The potential exposure to cyanotoxins during HABs can occur in drinking water when processing facilities are overwhelmed or in recreational waters from occupational and leisure activities. The health impact of these chronic, low-dose exposures remains an active and emerging topic of research, augmented by the potential of climate change to drive future algal blooms. In our current study, we successfully demonstrated a histopathologic promotion of hepatocellular lesions toward a more malignant phenotype (hepatocellular carcinoma in the *Microcystis* lysate exposure group). When combined with increased mortality rates due to *M. aeruginosa* lysate consumption and our previous findings detailing shifts in gut microbiome communities, considerable health status impacts could be associated with low-concentration chronic cyanobacterial and cyanotoxin ingestion [34].

## 4. Conclusions

Here, we show that the chronic ingestion of drinking water containing low doses of cyanotoxins successfully recapitulates an environmentally relevant delivery route and coordinates a harmful health outcome (a shift to increasingly malignant lesions) in pre-initiated mice. While the impact of low-dose MC cyanotoxins in healthy populations may remain limited, at-risk populations may be particularly susceptible to adverse health outcomes following these chronic exposures. Further investigation is essential to explore and understand the pathological mechanisms attributed to the combinatorial exposure to cyanotoxins, their metabolites, and other bioactive cellular compounds present during HABs.

## 5. Methods

### 5.1. Experimental Design

The *Microcystis aeruginosa* cultivation, animal husbandry, and experimental conditions for the current study are outlined in our previous publication [34]. Briefly, purified MC-LR (99%) was obtained from a commercial supplier (Beagle Bioproducts, Columbus, OH, USA), while *M. aeruginosa* were cultured, removed from suspension via centrifugation, and lysed to create a concentrated whole cell lysate. Single-use weekly aliquots of each cyanotoxin source were stored at −86 °C until needed and diluted to an ELISA-verified 10 µg/L MC-LR or total MCs. *Microcystis* cyanotoxin sources were administered *ad libitum* in drinking water to mice. At four weeks of age, a single intraperitoneal injection of diethylnitrosamine (DEN) was administered at 90 mg/kg body weight to 96 C3H/HeJ male mice to initiate liver carcinogenesis (Figure 5). Following a 3-week recovery period, experimental MC treatments were administered *ad libitum* in drinking water. Following 30 weeks of treatment (at 37 weeks of age), all surviving animals were euthanized and necropsied, and the tissue samples were collected.

### 5.2. M. aeruginosa Whole Cell Lysate Characterization

A sample of the *M. aeruginosa* lysate was analyzed via liquid chromatography-mass spectrometry (LC-MS) to define the chemical composition of the complex mixture. The sample was additionally screened for the presence of the neurotoxin β-N-methylamino-L-alanine (BMAA) using the β-N-methylamino-L-alanine (BMAA) ELISA colorimetric immunoassay kit (Abraxis BioScience #520040). *M. aeruginosa* used in this study was originally isolated from western Lake Erie in Ohio, USA and was purified and identified in our previous study using a Nanopore DNA sequencer (real-time sequencing technique; Oxford Nanopore Technologies, Oxford, UK) [35].

### 5.3. Microcystin Adsorption to Plastic Assessment

To study the potential loss of MC via plastic bottle adsorption over the course of a week, a 7-day experiment was conducted to monitor changes in MC concentrations. Clear plastic drip bottles obtained from The Ohio State University-University Laboratory Animal Resources (OSU-ULAR) were wrapped in foil and filled with reverse osmosis (RO) water containing 10 µg/L MC-LR or a cyanobacterial lysate containing 10 µg/L total MCs. The bottles were stored at room temperature with a 12 h light/dark cycle to replicate a typical week of toxin storage in animal cage environments. The samples were collected daily and stored at −20 °C in amber glass vials. Total MC concentrations were determined using the Abraxis BioScience ELISA kit, as previously described.

### 5.4. Gross and Histopathological Examination

At necropsy, the whole livers were surgically excised, weighed, and examined for gross lesions. If present, lesions were documented, enumerated, and measured (diameter). Following the gross examination, the liver samples were sectioned, placed in pathology cassettes, and fixed in 10% neutral buffered formalin for 24 h. The fixed liver samples were subsequently embedded in paraffin, sectioned, and stained using hematoxylin-eosin. Histopathological examination was conducted by a board-certified veterinary pathologist at the OSU Comprehensive Cancer Center Comparative Pathology and Mouse Phenotyping Shared Resource. The lesion counts and classifications were normalized to the surface area of the tissue observed using ImageJ software (National Institutes of Health, Bethesda, MD, USA, Version 1.52d) [36].

### 5.5. Statistical Analyses

All statistical analyses were conducted using Stata 10.4 software (StataCorp, LLC; College Station, TX, USA). Time series data were examined using linear regression methodology, lesion counts were analyzed using Kruskal–Wallis one-way analysis of variance, and lesion proportions were analyzed using a two-sided t-test for proportions. A Kaplan–Meier survivability analysis was conducted to analyze longitudinal mortality rates.

## Figures and Tables

**Table 1:** Lesion count and incidence as determined by gross examination. No significant differences in gross lesion counts were found across groups ^1^.

Group	Lesion Incidence	Gross Lesions per Mouse (Mean ± S.D.)
Water	21/30 (70%)	1.23 ± 0.27
MC-LR (10 µg/L)	9/22 (41%)	0.68 ± 0.21
Lysate (10 µg/L Total MCs)	10/15 (67%)	2.60 ± 0.93

^1^ Lesion density within the PB500 group was too high for gross enumeration and pathological classification; therefore, these data have been excluded from Table 1.

**Table 2:** Mean lesion counts per area of tissue (lesion count/mm^2^) ^1,2^.

Group	Preneoplastic Foci	Adenoma	Hepatocellular Carcinoma	Total Lesions
Water	0.018 ± 0.028	0.004 ± 0.018	0.003 ± 0.008	0.026 ± 0.034
MC-LR (10 µg/L)	0.008 ± 0.012	0.001 ± 0.003	0.007 ± 0.011	0.015 ± 0.019
Lysate (10 µg/L Total MCs)	0.015 ± 0.019	0.004 ± 0.009	0.023 ± 0.058	0.042 ± 0.068

^1^ Lesion density within the PB500 group was too high for gross enumeration and pathological classification; therefore, these data have been excluded from Table 2. ^2^ All values reported as “mean ± s.d”.

## Data Availability

The data presented in this study are available on request from the corresponding author.

## Supplementary Materials

The following supporting information can be downloaded at: https://www.mdpi.com/article/10.3390/toxins14070484/s1, Figure S1: Chemical composition of *M. aeruginosa* Lysate as determined via LC-MS/MS. Proportions of MCs in the cyanobacterial Lysate were as follows: MC-RR (37%); [D-Asp3] MC-RR (24%); MC-LR (22%); and MC-YR (17%); Figure S2: Adsorption of microcystins onto drip bottle plastic. We noted no significant loss or adsorption of MC-LR or total MCs in the cyanobacterial Lysate in the drip bottles, over a 7-day period. Bars indicate standard deviation; Figure S3: Mean mouse weight over study duration. There were no significant differences in mouse weight across treatment groups. Bars indicate standard deviation; Figure S4: Mean water consumption, per mouse, over study duration. We noted significantly lower (*p* < 0.001) water consumption in the positive control (PB500) group compared to other treatments. When comparing MC treatments to water control, there were no significant differences in water consumption across the groups. Bars indicate standard deviation.
