# Supplementary material for: Impact of Cyanotoxin Ingestion on Liver Cancer Development Using an At-Risk Two-Staged Model of Mouse Hepatocarcinogenesis
Source: Toxins (Basel). 2022 Jul 14;14(7):484. doi: 10.3390/toxins14070484 (PMC9320861; doi:10.3390/toxins14070484)
Supplement: Supplementary file 1 [file toxins-14-00484-s001.zip › toxins-1784688-supplementary.pdf]

Supplementary Figures: Impact of Cyanotoxin Ingestion on Liver Cancer Development Using an At-Risk Two-Staged Model of Mouse Hepatocarcinogenesis

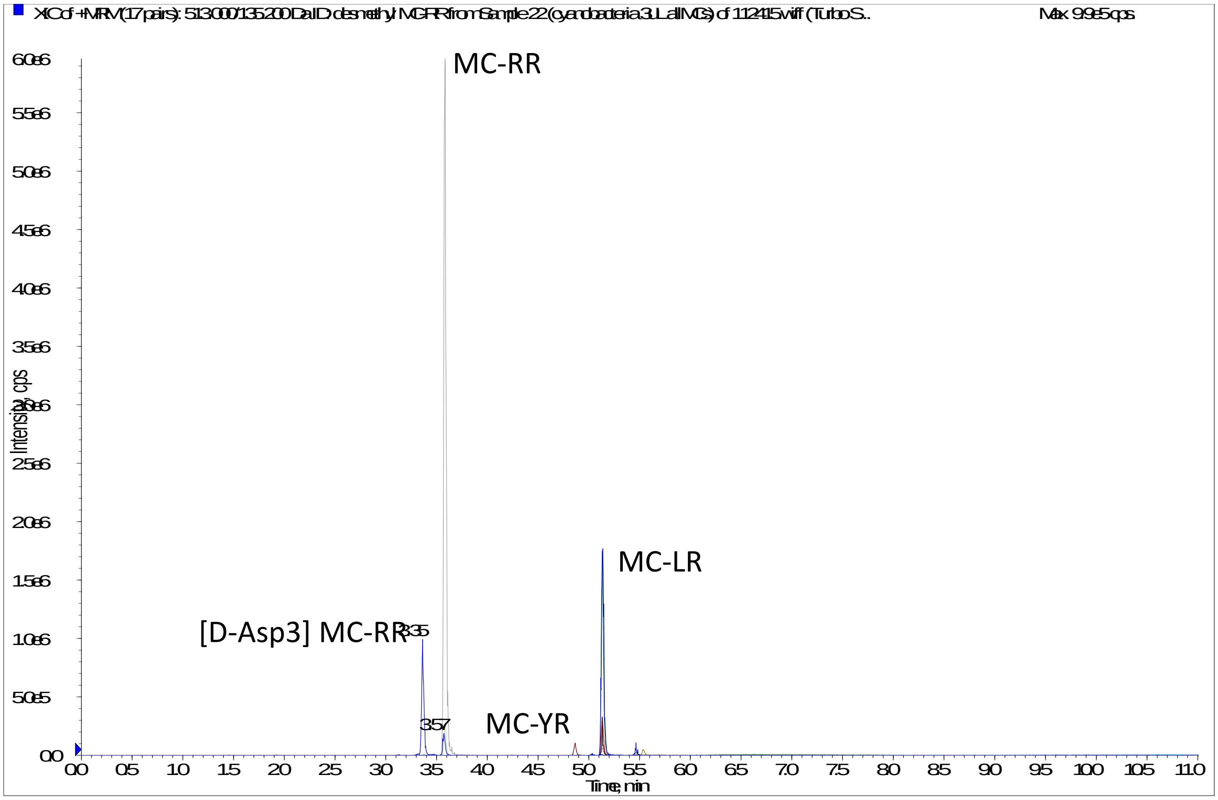

Figure S1. Chemical composition of *M. aeruginosa* Lysate as determined via LC-MS/MS. Proportions of MCs in the cyanobacterial Lysate were as follows: MC-RR (37%); [D-Asp3] MC-RR (24%); MC-LR (22%); and MC-YR (17%).

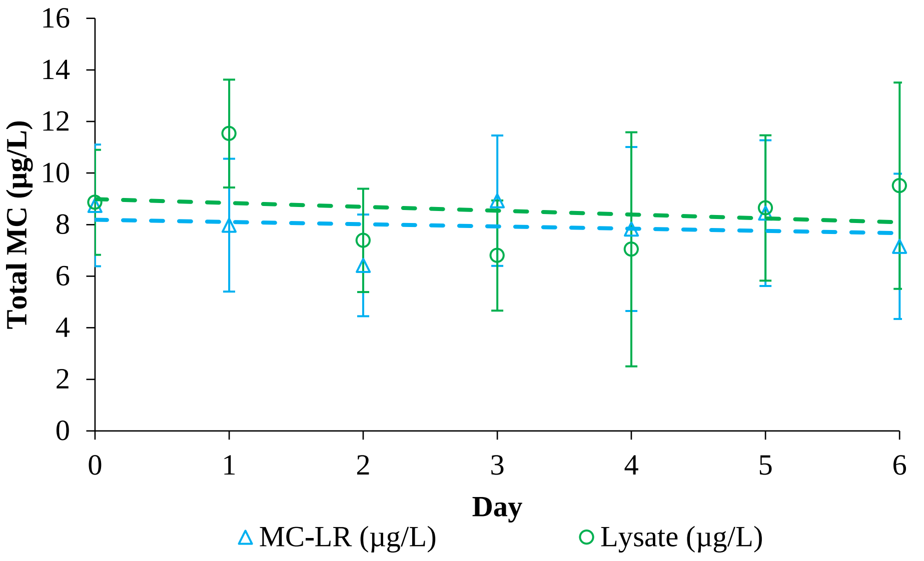

Figure S2. Adsorption of microcystins onto drip bottle plastic. We noted no significant loss or adsorption of MC-LR or total MCs in the cyanobacterial Lysate in the drip bottles, over a 7-day period. Bars indicate standard deviation.

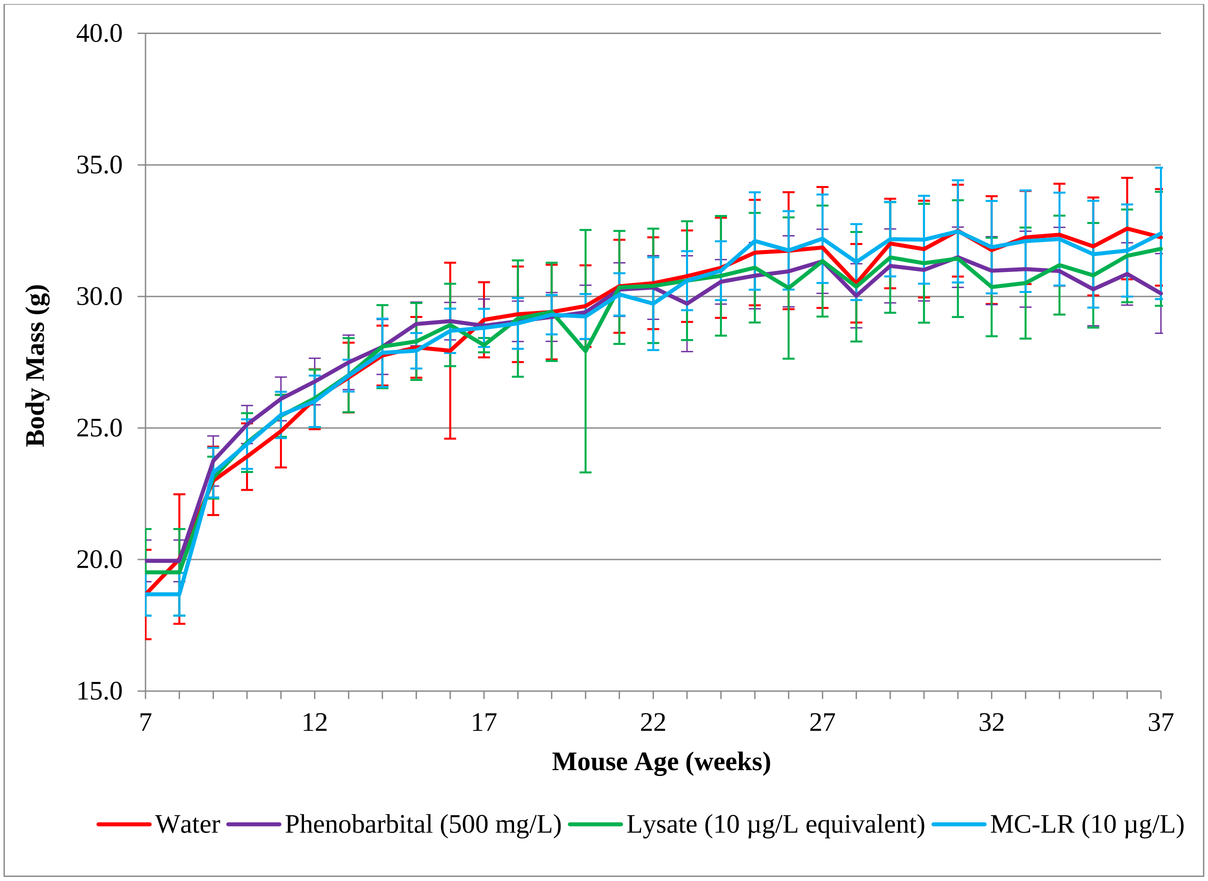

Figure S3. Mean mouse weight over study duration. There were no significant differences in mouse weight across treatment groups. Bars indicate standard deviation.

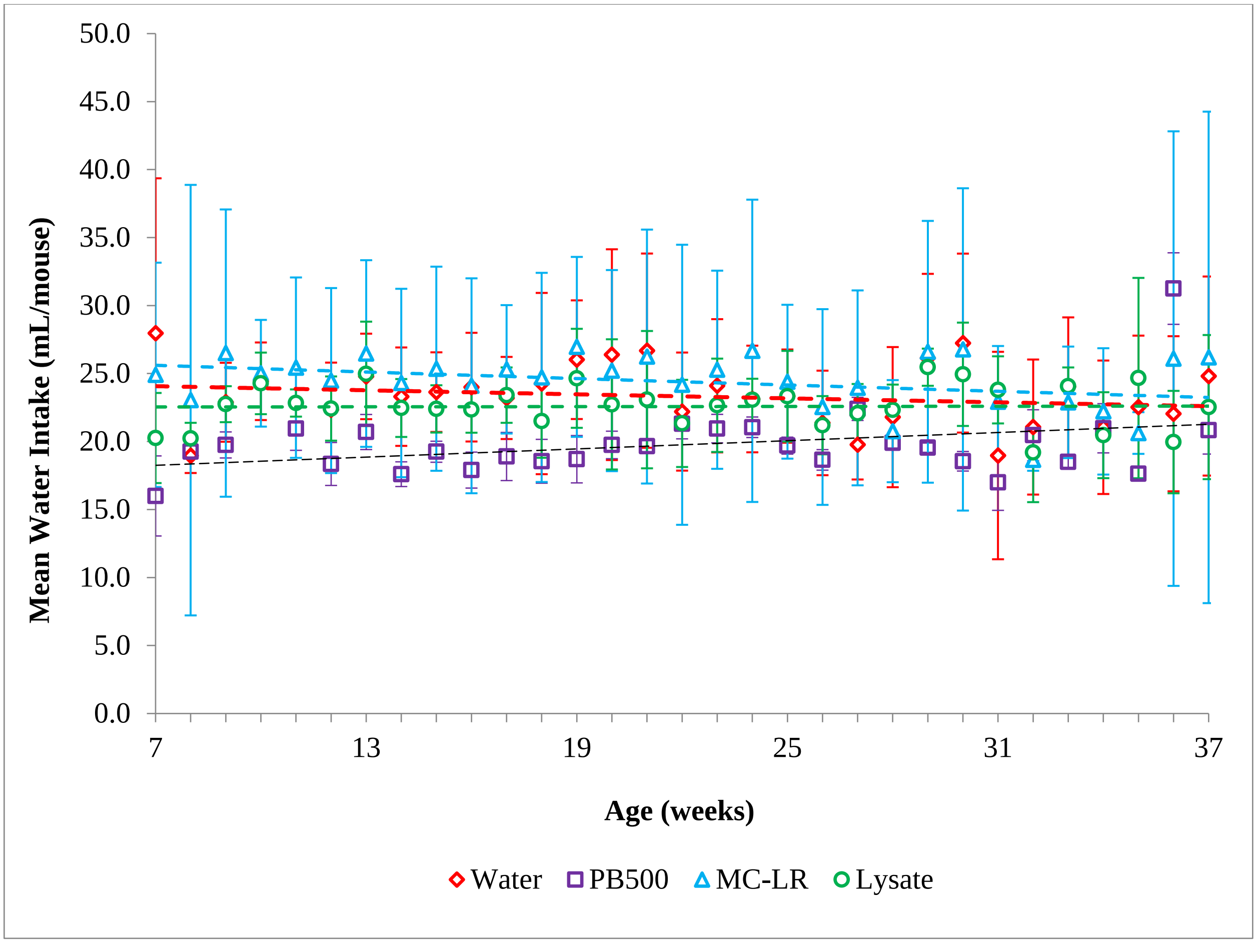

Figure S4. Mean water consumption, per mouse, over study duration. We noted significantly lower (p < 0.001) water consumption in the positive control (PB500) group compared to other treatments. When comparing MC treatments to water control, there were no significant differences in water consumption across the groups. Bars indicate standard deviation.
